# Brazilian adaptation and validation of the Multidimensional Measure of Parasocial Relationships (MMPR)

**DOI:** 10.1038/s41598-025-21187-z

**Published:** 2025-10-27

**Authors:** Thiago Medeiros da Costa Daniele, Letticia de Araújo Moura, Milgen Sánchez-Villegas, Elina Björk, Danilo Garcia

**Affiliations:** 1 Postgraduate Program in Public Health (PPGSC), University of Fortaleza (UNIFOR), Fortaleza, CE Brazil; 2 Promotion of Health and Innovation (PHI) Lab, International Network for Well-Being, Linköping, Sweden; 3 Promotion of Health and Innovation for Well-Being (PHI-WELL) Research Group, Department of Social Studies, University of Stavanger, Stavanger, Norway; 4 University of Fortaleza (UNIFOR), Fortaleza, CE Brazil; 5 Facultad de Ciencias Jurídicas y Sociales, Centro de Investigación e Innovación Social José Consuegra Higgins, Universidad Simón Bolívar, Barranquilla, Colombia; 6 Department of Behavioral Sciences and Learning, Linköping University, Linköping, Sweden; 7 Department of Social Studies, University of Stavanger, Stavanger, Norway; 8 Lab for Biopsychosocial Personality Research (BPS-PR), International Network for Well-Being, Stavanger, Norway; 9 Centre for Ethics, Law and Mental Health (CELAM), University of Gothenburg, Gothenburg, Sweden; 10 Department of Psychology, University of Gothenburg, Gothenburg, Sweden

**Keywords:** Brazil, Confirmatory factor analysis (CFA), Multidimensional measure of parasocial relationships (MMPR), Parasocial relationships, Psychometrics, Social media engagement, Psychology, Human behaviour

## Abstract

Social media has intensified parasocial relationships, one-sided bonds between individuals and media figures. While extensively researched in Western populations, parasocial engagement remains unexplored in culturally diverse contexts, particularly Latin America. The Multidimensional Measure of Parasocial Relationships (MMPR) is a validated tool that captures four dimensions of parasocial relationship engagement: Affective, Cognitive, Behavioral, and Decisional. Cross-cultural validation of parasocial relationship measures is essential for understanding how cultural context shape media psychology phenomena. We aimed to validate the Brazilian Portuguese adaptation of the MMPR, with four primary objectives: (1) replicate the correlated bifactor structure from previous research, (2) evaluate internal consistency and dimensional performance in the Brazilian cultural context, (3) analyze the intercorrelations among those dimensions, and (4) examine measurement invariance across gender. Brazilian participants (*N* = 398; *M*_age_ = 24.66, *SD* = 9.24) completed the 18-item Brazilian MMPR. We conducted a Confirmatory Factor Analysis (CFA) using the Weighted Least Square Mean and Variance method to test the correlated bifactor model. Internal consistency was evaluated using *Cronbach’s Alpha* (α) and *McDonald’s Omega* (ω), with Spearman correlations examining inter-dimensional relationships. Additionally, multi-group confirmatory factor analysis was conducted to assess measurement invariance across gender. CFA supported the correlated bifactor structure with acceptable fit indices (*χ²* = 190.89, *RMSEA* = 0.08, *CFI* = 0.91). Standardized loadings ranged from − 0.40 to 0.79. The total scale demonstrated high internal consistency (α/ω = 0.88/0.87), while coefficients varied across dimensions (Affective: α/ω = 0.64/0.66; Cognitive: α/ω = 0.75/0.75; Behavioral: α/ω = 0.58/0.58; Decisional: α/ω = 0.82/0.81). The Decisional dimension demonstrated the strongest correlation with overall parasocial engagement (ρ = 0.86), whereas the Behavioral dimension showed the weakest (ρ = 0.65). Measurement invariance testing showed acceptable model fit for women, consistent with the full sample; however, convergence issues for the male subsample prevented further assessment of configural invariance. The Brazilian adaptation of the MMPR replicated the correlated bifactor structure and demonstrated strong overall reliability, supporting its uses as a valid measure of parasocial engagement in Brazilian populations. However, variability in the reliability of individual dimensions, particularly the strong contribution of the Decisional dimension and the weaker performance of the Behavioral dimension, suggest culturally specific patterns in parasocial engagement. Additionally, partial evidence for gender invariance suggests the scale performs more consistently among women than men, warranting further investigation. These findings highlight the need for culturally sensitive psychometric adaptations and provide a foundation for future cross-cultural research in consumer behavior, social media psychology, and therapeutic applications.

## Introduction

The rise of social media and streaming platforms has fundamentally transformed parasocial dynamics by decreasing the perceived proximity to public figures. Parasocial relationships, non-reciprocal emotional bonds between media users and figures such as influencers, celebrities, or fictional characters^1–3^, have gained increasing importance as individuals engage across platforms like Instagram, TikTok, Facebook, etcetera^4^. These asymmetrical relationships evoke feelings of intimacy, understanding, and friendship that parallel real-world social connections while lacking reciprocal engagement^1,5^. Since their initial conceptualization^1^, parasocial relationships have evolved from passive, one-sided interactions primarily associated with traditional media (e.g., television, radio) to dynamic processes central to digital platforms. Modern parasocial relationships are shaped by the increased accessibility and perceived authenticity of media figures^5^ with digital platforms amplifying these dynamics through real-time interactions such as comments and “likes” that blur boundaries between mediated and real-world social connections^3,6,7^. This creates a false sense of intimacy with media figures^8,9^.

Parasocial relationships have significant implications for emotional well-being, social connection, and identity development. These connections can serve as coping mechanisms during periods of loneliness or social isolation, providing individuals with a sense of companionship and emotional support^6^. Social media platforms intensify these effects by offering more frequent and personalized glimpses into media figures’ lives, enhancing perceived authenticity and relatability^7^. However, outcomes are not universally positive; parasocial relationships can for example exacerbate social comparison and unrealistic expectations, leading to body dissatisfaction and reduced self-esteem^10,11^. Moreover, previous research suggests that gender differences may influence parasocial engagement^2,8^. These contrasting effects underscore the importance of contextual and cultural factors in shaping the impact of parasocial engagement.

Despite expanding research, there remains a critical lack of investigation in non-western contexts, as noted by Ghai and colleagues^4^, who highlight the overrepresentation of Global North populations and insufficient exploration of Global South contexts. This gap is particularly evident in regions like Brazil, were distinct media consumption patterns and sociocultural norms shape interactions with media figures in unique ways. Brazilian audiences are avid users of Instagram, Facebook, WhatsApp, and YouTube, creating opportunities for strong parasocial connections with both local and global influencers^12^. With Internet penetration reaching 86.6% of the total population by early 2024 ^31^, Brazilians use these platforms for communication, news consumption, entertainment, and building parasocial relationships with digital influencers^13^. However, the absence of local studies limits our understanding of how these relationships develop and their implications for emotional well-being and social behavior in such contexts. Before exploring these culturally specific dynamics, establishing reliable and valid measurement tools adapted to the Brazilian context is essential.

Indeed, a cornerstone of understanding parasocial relationships lies in the ability to measure their multifaceted nature. Horton and Wohl^1^, who first conceptualized parasocial relationships, identified three core features: (1) emotional connection based on the illusion of friendship, intimacy, and affection arising from repeated exposure, (2) perceived familiarity that creates the belief of genuinely knowing the media figure, and (3) identification through aligning with the attitudes and behaviors of the media figure. In digital contexts, these foundational features have evolved significantly. Perceived similarity, whether in lifestyle, values, or beliefs, plays a central role in the formation and maintenance of parasocial bonds. Users engage in symbolic acts such as “liking,” commenting, or sharing, which, while not constituting real interaction, foster an illusion of mutual exchange and involvement. Building on this framework, the Multidimensional Measure of Parasocial Relationships (MMPR) was developed by Garcia and colleagues^3^ to operationalize parasocial engagement through four dimensions:

A. Affective: Emotional bonds such as admiration, attachment, or affection toward media figures.
B. Behavioral: Mediated interactions through social media platforms, such as “liking”, commenting, or sharing content.
C. Cognitive: Mental engagement including thinking about, analyzing, or relating to media figures’ characteristics, values, or life events.
D. Decisional: Perceived influence of media figures on daily decisions, including lifestyle choices, consumption habits, and personal aspirations.

In their original study, Garcia and colleagues^3^ identified the correlated bifactor model as the most appropriate representation of the MMPR’s structure, integrating a general factor representing overall parasocial engagement alongside the four specific factors corresponding to the Affective, Behavioral, Cognitive, and Decisional dimensions (i.e., the ABCD Model of parasocial relationships engagement). The bifactor structure seems to capture the dual nature of parasocial relationships by reflecting a general engagement tendency while preserving distinct dimensional characteristics. The interconnected, yet distinct factors, highlight the roles of emotional connections, cognitive processes, social media interactions, and perceived decision-making influence, providing a comprehensive framework that moves beyond traditional unidimensional measures. Moreover, the MMPR has demonstrated significant associations with mental health and well-being. Higher levels of parasocial engagement, particularly those captured by the general MMPR score, appear positively associated with the tendency to compare oneself to others, which in turn leads to low levels of self-esteem^3^.

### The present study

The present study aimed to investigate whether the MMPR, as originally developed by Garcia and colleagues^3^, maintains it psychometric integrity when adapted to a Brazilian cultural context. Specifically, we sought to (1) test whether the correlated bifactor structure found by Garcia and colleagues in their Swedish sample^3^, comprising a general parasocial engagement factor and four specific dimensions (Affective, Cognitive, Behavioral, and Decisional), would be supported in a Brazilian sample through Confirmatory Factor Analysis (CFA). This bifactor structure, validated in Hungary, Indonesia, Canada, and Poland in recent studies^14,15^, conceptualizes parasocial relationships as both a unified and multidimensional construct, allowing for a nuanced assessment of individuals’ engagement with media figures. Our central hypothesis was that the MMPR’s bifactor model would demonstrate acceptable fit indices in Brazil, indicating its cross-cultural applicability. We further hypothesized that the general factor would account for a substantial proportion of the variance, while each specific factor would contribute uniquely to the measurement of parasocial engagement. Additionally, we aimed to (2) assess the reliability of the Brazilian adaptation of the MMPR by examining the internal consistency of its dimensions and (3) analyze the intercorrelations among those dimensions. Given Brazil’s distinctive digital media landscape, we hypothesize that some MMPR items and dimensions may exhibit culturally specific patterns of performance, indicating the potential need for future refinement to enhance the scale’s cultural sensitivity and validity. Additionally, multi-group CFA was conducted to (4) assess measurement invariance across gender, evaluating the stability of the factorial structure between men and women. By establishing the MMPR’s reliability and cultural relevance, this study provides a validated tool for future research and contribute to a broader understanding of how parasocial relationships manifest across diverse sociocultural landscapes. These findings will inform both theoretical advancements and practical interventions tailored to the Brazilian context, thus addressing a key gap in the current literature.

## Methods

### Ethical statement

The data were collected through voluntary and anonymous participation following ethical guidelines established by the Declaration of Helsinki and Brazilian regulations for research involving human subjects, specifically CNS Resolution No. 466/12 (*Conselho Nacional de Saúde*, 2012) and the complementary Resolution No. 510/16 for humanities and social sciences research^16^. The study received approval from a Brazilian Research Ethics Committee (Protocol No. 6.093.64) and adhere to the General Data Protection Law (LGPD, Law No. 13,709/2018) for data confidentiality and participant privacy^17^. Informed consent was obtained from all participants prior data collection.

### Procedure and participants

This study employed a methodological design focused on the translation, cultural adaptation and validation of the MMPR from English into Brazilian Portuguese. The adaptation process followed internationally recognized guidelines for cross-cultural adaptation of self-report measures^18,19^. Translation procedures involved forward, and backward translation processes conducted by bilingual experts and native Brazilian Portuguese speakers to ensure linguistic accuracy and conceptual equivalence. A committee of experts in survey methodology reviewed the preliminary translated version to refine the final MMPR Brazilian adaptation.

Participants aged 18 and above and actively engaged on at least one social media platform were eligible for inclusion. The study employed a virtual snowball sampling method to reach specific populations through online networks and referrals^20^. Participants were encouraged to refer colleagues and friends, facilitating an expansive reach for a diverse validation sample. A total of 517 participants initially responded to the survey; however, 119 (23%) did not complete all items, resulting in a final sample of 398 participants (192 women, 206 men) with an age mean of 24.66 years (*SD* = 9.24).

### Measure

#### Engagement in parasocial relationships

The MMPR was developed by Garcia and colleagues^3^ to operationalize parasocial engagement as a multidimensional construct grounded in attitude theory. The instrument examines individuals’ levels of engagement with social media figures through four theoretically driven, interconnected dimensions: Affective (A), Behavioral (B), Cognitive (C), and Decisional (D). The MMPR comprises 18 items (4–6 items per scale) rated on a 4-point Likert scale ranging from 1 (*Totally Disagree*) to 4 (*Totally Agree*). Participants receive detailed instructions to select a specific social media figure they follow (e.g., influencer, YouTuber, TikToker) and with whom they have no real-life relationship (see Table 1 for complete instructions and items in English and Brazilian Portuguese). Garcia and colleagues’ original validation study^3^ demonstrated acceptable internal consistency (*Cronbach’s Alpha* = 0.66-0.75 for dimensions, 0.85 for total scale) and confirmed the correlated bifactor model as the optimal factor structure (CFI = 0.95, RMSEA = 0.07).

**Table 1:** The multidimensional measure of parasocial relationships (MMPR) in english and Brazilian Portuguese.

	English	Brazilian Portugues	
Instructions	This questionnaire contains statements about your attitudes, that is, your thoughts, feelings, and behavior regarding a specific social media figure. Before giving your answers, please think of a specific social media figure, preferably the one that you follow the most. It can be an influencer, a youtuber, tiktoker, or a social media figure within, for example, lifestyle, exercise, sports, gaming, nutrition, or fashion. The most important thing is that you don’t have a relation with this person in real life. Please answer to which degree you agree or disagree to the following statements:	Este questionário contém afirmações sobre suas atitudes, como por exemplo: seus pensamentos, sentimentos e comportamentos referentes a uma pessoa pública específica que você acompanha nas mídias sociais. Antes de marcar suas respostas, pense em uma pessoa pública, de preferência uma que você mais acompanha nas redes sociais. Pode ser um(a) influenciador(a), um(a) youtuber, um(a) tiktoker ou uma pessoa que você segue e cria conteúdos nas áreas de estilo de vida, exercícios físicos, esportes, jogos, alimentação ou moda. O mais importante que você deve considerar ao responder esse questionário é o fato de não haver nenhuma relação real com a pessoa pública escolhida. Por favor, responda em que grau você concorda ou discorda com as seguintes afirmações:	Instruções
Affective Dimension	I experience a feeling of connectedness with the media figure through his/her posts on social media.	Eu tenho um sentimento de conexão com a pessoa pública que eu acompanho através das suas postagens.	Dimensão Afetiva
	I experience emotional engagement when the social media figure shares more private information about himself/herself (e.g., bigger life events).	Eu sinto que me envolvo emocionalmente quando a pessoa pública compartilha informações privadas da sua vida (por exemplo: problemas pessoais, familiares e/ou financeiros).	
	I don’t feel like I can personally relate to the content in the social media figure´s posts.	Sinto que os conteúdos postados por essa pessoa pública não se relacionam com minha vida.	
	I often feel that I get inspired by the social media figure´s posts.	Eu frequentemente sinto que sou inspirado(a) pelas postagens dessa pessoa pública.	
Cognitive Dimension	I think that the social media figure represents values that are important to me.	Eu penso que essa pessoa pública representa valores importantes para mim.	Dimensão Cognitiva
	I don’t think that the media figure portrays himself/herself in an authentic way on social media.	Acho que essa pessoa pública não se mostra de maneira real nas redes sociais.	
	I see most of what the media figure shares on social media in a positive way.	Eu concordo com a maioria das postagens que essa pessoa pública compartilha nas redes sociais.	
	The social media figure seems to be a genuine person that I would get along with in real life.	Essa pessoa pública parece ser autêntica a ponto de eu querer ter uma relação com ela (ele) na vida real.	
Behavior Dimension	I always “like” the media figure’s posts on social media.	Eu sempre “curto” as postagens dessa pessoa pública nas redes sociais.	Dimensão Comportamental
	I often comment on the social media figure´s posts in the comment field.	Eu frequentemente faço comentários nas postagens dessa pessoa pública.	
	I often forward the social media figure´s posts to my friends or share them on my own online feeds.	Eu frequentemente costumo encaminhar as postagens dessa pessoa pública para meus amigos e/ou compartilho nas minhas redes sociais.	
	I mostly just check the social media figure´s updates and am not that active with liking, sharing, or commenting.	Na maioria das vezes, apenas verifico as atualizações da pessoa pública e não sou tão ativo com curtidas, compartilhamentos ou comentários.	
Decisional Dimension	I prefer things that the social media figure is marketing (e.g., products, nutrition advice, training advice, etc.) over similar things that are marketed in other places.	Tenho preferência pelos produtos que essa pessoa está divulgando (por exemplo, conselhos nutricionais, conselhos de treinamento, etc.) quando comparado aos produtos divulgados em outros locais.	Dimensão Decisional
	The social media figure´s posts often inspire me to make changes in my own life.	As publicações dessa pessoa pública geralmente me inspiram a fazer mudanças na minha vida.	
	I never buy products that the media figure is marketing or giving advice about on social media.	Eu nunca compraria produtos que essa pessoa pública está divulgando ou aceitaria conselhos que ele/ela compartilha nas redes sociais.	
	I happily follow different tips and advice that the social media figure shares because I feel I can trust his/her knowledge about these things.	Eu sigo prazerosamente as diferentes dicas e conselhos que essa pessoa pública compartilha por sentir que posso confiar naquilo que ele/ela divulga.	
	It often happens that, in conversations with other people in my everyday life, I point out things that the media figure has mentioned in his/her posts on social media.	Em conversas com outras pessoas do meu dia a dia, eu aponto coisas que essa pessoa pública divulga ou posta em suas redes sociais.	
	There are times when the social media figure´s posts contribute to me changing my lifestyle in some way (e.g., clothing, diet, training routine, looks, and etc.).	As postagens dessa pessoa pública contribuem para que eu, de alguma forma, mude meus hábitos de vida (por exemplo: roupas, dieta, rotina de treinos, aparência, etc.).	

Originally published in Björk, E. (2021). Parasociala Relationer som Attityd till Mediaprofiler i Sociala Medier – Komponenter i attitydbildning och dess samband med social jämförelse och självkänsla [Bachelor Thesis, Linköping University]. See: Garcia, D., Björk, E., & Kazemitabar, M. (2022). The A(ffect) B(ehavior) C(ognition) D(ecision) of Parasocial Relationships: A Pilot Study on the Psychometric Properties of the Multidimensional Measure of Parasocial Relationships (MMPR). Heliyon, 8:e10779. 10.1016/j.heliyon.2022.e10779. Translation to Brazilian Portuguese by Thiago M. da C. Daniele, PhD, and Matheus E. Van Tol Amaral Guerra (2023). For any use, research or commercial, please contact danilo.garcia@icloud.com and elina.bjork@icloud.com.

### Data analyses

All analyses were conducted using RStudio (RStudio Team, Version 2024.04.2 + 764; https://posit.co/download/rstudio-desktop/) with the R programming language (R Core Team, Version 4.4.1; https://www.r-project.org/). Descriptive statistics were computed for all items, and assumptions of normality were assessed using skewness and kurtosis indices. Several items exhibited non-normal distributions, which is common for Likert-type scales. Given these findings and the ordinal nature of the data, non-parametric procedures were prioritized where appropriate.

We used a CFA to test the correlated bifactor model initially proposed and validated by Garcia and colleagues^3^ in a Swedish sample, and subsequently replicated in Hungary, Indonesia, Canada, and Poland^14,15^. This model includes a general factor representing overall parasocial engagement, along with the four specific factors: Affective, Behavioral, Cognitive, and Decisional. The model was estimated using robust maximum likelihood estimation, which is appropriate for ordinal data and deviations from normality. Internal consistency of the MMPR and its subscales was evaluated using both *Cronbach’s Alpha* (α) and *McDonald’s Omega* (ω) coefficients. *McDonald’s Omega* was included to provide a more robust reliability estimate, addressing the *Cronbach’s Alpha*’s limitations under conditions of unequal item loadings or non-tau-equivalence. Correlations among the MMPR dimensions and the total score were examined using Spearman’s rho (*ρ*) due to the ordinal response format and non-normal data distribution. This methodological choice ensures the validity and robustness of our correlation analyses given our data characteristics.

Measurement invariance across gender was examined using a multi-group CFA approach. Configural invariance was assessed by testing whether the same factor structure holds across gender groups. Due to the correlated bifactor model complexity, invariance testing followed a stepwise approach, beginning with configural invariance before proceeding to metric and scalar invariance if model convergence permitted. Model comparison was planned using chi-square difference tests and changes in fit indices (ΔCFI ≤ 0.01, ΔRMSEA ≤ 0.015) as decision criteria for invariance acceptance.

## Results

### Confirmatory factor analysis (CFA)

The CFA indicated acceptable model fit: *χ² = 190.891*,* df = 111*,* p < .001; Satorra-Bentler χ² = 360.649*,* df = 111*,* p < .001*. Although the chi-square test was significant, likely due to the large sample size, other fit indices supported the adequacy of the correlated bifactor model (*RMSEA* = 0.08; *CFI* = 0.91; *SRMR* = 0.05), consistent with the initial findings by Garcia and colleagues^3^. Standardized factor loadings revealed mixed results. For the general factor, ten items showed positively significant standardized loadings, with seven items meeting the 0.40 threshold (ranging from 0.41 to 0.62). For the specific factors, loadings ranged from − 0.40 to 0.79, with most being statistically significant (*p* < .001). However, two items fell below the 0.40 threshold: one from the Affective dimension (MMPR03 = 0.036) and one from the Behavioral dimension (MMPR12 = 0.36), indicating weaker associations with their respective factors. Items within the Decisional dimension displayed negative loadings, though all exceeded − 0.40. Covariances among the specific factors were significant (*p* < .001), ranging from 0.70 to 0.79, except for the Decisional dimension, which showed significant but negative associations (-0.74 to − 0.78) with all other dimensions (see Table 2).

**Table 2:** Factor loadings and estimates for the Brazilian version of the multidimensional measure of parasocial relationships (MMPR).

Factors	Items	Estimate	SE	Z	*P*	Variance	Std. estimations
Affective dimension	MMPR01	0.79	0.038	20.925	> 0.001	0.790	0.790
	MMPR02	0.614	0.042	14.489	> 0.001	0.614	0.614
	MMPR03	0.36	0.051	7.106	> 0.001	0.360	0.360
	MMPR04	0.572	0.095	6.001	> 0.001	0.572	0.572
Cognitive dimension	MMPR05	0.713	0.075	9.444	> 0.001	0.713	0.713
	MMPR06	0.631	0.042	15.109	> 0.001	0.631	0.631
	MMPR07	0.669	0.055	12.252	> 0.001	0.669	0.669
	MMPR08	0.674	0.053	12.635	> 0.001	0.674	0.674
Behavioral dimension	MMPR09	0.685	0.056	12.322	> 0.001	0.685	0.685
	MMPR10	0.702	0.054	13.101	> 0.001	0.702	0.702
	MMPR11	0.608	0.043	14.254	> 0.001	0.608	0.608
	MMPR12	0.360	0.078	4.593	> 0.001	0.360	0.360
Decisional dimension	MMPR13	-0.498	0.078	-6.356	> 0.001	− 0.498	− 0.498
	MMPR14	-0.577	0.105	-5.468	> 0.001	− 0.577	− 0.577
	MMPR15	-0.401	0.054	-7.389	> 0.001	− 0.401	− 0.401
	MMPR16	-0.773	0.064	-12.056	> 0.001	− 0.773	− 0.773
	MMPR17	-0.689	0.042	-16.319	> 0.001	− 0.689	− 0.689
	MMPR18	-0.529	0.103	-5.142	> 0.001	− 0.529	− 0.529
MMPR total score	MMPR01	0.150	0.118	1.271	0.204	0.150	0.150
	MMPR02	0.128	0.102	1.263	0.206	0.128	0.128
	MMPR03	-0.004	0.077	-0.048	0.961	− 0.004	− 0.004
	MMPR04	0.625	0.079	7.904	> 0.001	0.625	0.625
	MMPR05	0.503	0.102	4.943	> 0.001	0.503	0.503
	MMPR06	-0.064	0.099	-0.647	0.518	− 0.064	− 0.064
	MMPR07	0.318	0.103	3.079	0.002	0.318	0.318
	MMPR08	0.274	0.103	2.670	0.008	0.274	0.274
	MMPR09	0.231	0.107	2.166	0.030	0.231	0.231
	MMPR10	-0.122	0.115	-1.064	0.287	− 0.122	− 0.122
	MMPR11	-0.006	0.097	-0.064	0.949	-0.006	-0.006
	MMPR12	-0.409	0.075	-5.429	> 0.001	− 0.409	− 0.409
	MMPR13	0.466	0.083	5.645	> 0.001	0.466	0.466
	MMPR14	0.684	0.092	7.473	> 0.001	0.684	0.684
	MMPR15	0.041	0.085	0.480	0.631	0.041	0.041
	MMPR16	0.409	0.120	3.417	0.001	0.409	0.409
	MMPR17	0.168	0.106	1.578	0.115	0.168	0.168
	MMPR18	0.650	0.086	7.598	> 0.001	0.650	0.650

### Internal consistency

The total scale demonstrated high internal consistency with a *Cronbach’s Alpha* (α) of 0.88 and a *McDonald’s Omega* (ω) of 0.87. At the dimensional level, internal consistency varied considerably. The Decisional dimension showed the strongest reliability (α = 0.82, ω = 0.81), followed by the Cognitive dimension (α = 0.75, ω = 0.75), which achieved acceptable consistency. The Affective dimension yielded moderate reliability (α = 0.64, ω = 0.66), falling below the conventional ≥ 0.70 threshold but remaining within acceptable ranges for exploratory research. The Behavioral dimension demonstrated the lowest reliability (α = 0.58, ω = 0.58), suggesting potential issues with item consistency or cultural interpretation of behavioral engagement measures.

### Bivariate inter-dimensional correlations

*Spearman’s rho* (*ρ*) correlation among the MMPR dimensions revealed distinct patterns of associations. The Affective dimension showed moderate correlation with the Behavioral dimension (*ρ* = 0.46), indicating a connection between emotional bonds and engagement behaviors. Stronger correlations emerged between the Affective and Cognitive dimensions (*ρ* = 0.59) and between the Affective and Decisional dimensions (*ρ* = 0.62), suggesting closer interplay between emotions, thoughts, and perceived influence. The Behavioral dimension demonstrated moderate correlations with both the Cognitive (*ρ* = 0.41) and Decisional (*ρ* = 0.37) dimensions, reflecting the link between engagement behaviors and higher-order cognitive processes. A strong correlation between the Cognitive dimension and Decisional dimensions (*ρ* = 0.66) highlighted the role of cognitive engagement in mediating perceived decision-making influence. Correlations with the total MMPR score revealed that the Decisional dimension had the strongest correlation (*ρ* = 0.86), followed by the Cognitive (*ρ* = 0.84) and Affective (*ρ* = 0.81) dimensions. The Behavioral dimension showed a comparatively weaker correlation (*ρ* = 0.65), consistent with its lower associations with the other dimensions (see Table 3).

**Table 3:** Correlation (*Spearman’s rho*) analyses among the dimensions of the Brazilian version of the multidimensional measure of parasocial relationships (MMPR).

	MMPR Affective Dimension	MMPR Behavioral Dimension	MMPR Cognitive Dimension	MMPR Decisional Dimension	MMPR Total Score
MMPR affective dimension	—				
MMPR behavioral dimension	0.457^**^	—			
MMPR cognitive dimension	0.595^**^	0.412^**^	—		
MMPR decisional dimension	0.625^**^	0.374^**^	0.658^**^	—	
MMPR total score	0.808^**^	0.652^**^	0.841^**^	0.865^**^	—

### Measurement invariance across gender

Measurement invariance testing across gender yielded mixed results due to estimation difficulties. For women (*n* = 192), the correlated bifactor model demonstrated acceptable fit: *χ²* = 146.669, *df* = 111, *p* < .001; *Satorra-Bentler χ²* =255.303, *df* = 111, *p* < .001, RMSEA = 0.099, CFI = 0.88, SRMR = 0.06. The factors loading pattern largely mirrored that of the full sample. Standardized loadings for specific factors were statistically significant (*p* < .001), ranging from 0.41 to 0.80, with the exception of one item in the Behavioral dimension (MMPR12 = 0.24), which fell below the 0.40 threshold. Items within the Decisional dimension again showed negative but significant loadings (ranging from − 0.46 to − 0.81). Covariances between Affective, Behavioral, and Cognitive dimensions were positive and significant (*p* < .001), ranging from 0.76 to 0.86, whereas the Decisional dimension showed significant negative covariances with all other dimensions (ranging from − 0.70 to -0.82). For men (*n* = 206), however, the model failed to converge properly due to estimation problems, including negative variances and improper solutions, which prevented adequate model identification.

## Discussion

The present study validated the Brazilian Portuguese adaptation of the MMPR by examining its factor structure, internal consistency, associations between MMPR dimensions, and measurement invariance across gender. In sum, the results provided support for the bifactor model, originally identified by Garcia and colleagues^3^, confirming the presence of a general factor of parasocial engagement alongside the four specific factors: Affective, Behavioral, Cognitive, and Decisional. Furthermore, the Brazilian MMPR demonstrated strong overall internal consistency, establishing it as a psychometrically sound tool for assessing parasocial relationships in this cultural context. However, dimensional performance varied considerably, revealing important cultural insights. The Decisional dimension showed the strongest reliability suggesting that perceived influence of media figures on personal decisions is particularly salient and consistently conceptualized among Brazilian participants. In contrast, the Behavioral dimension exhibited the weakest internal consistency, which may reflect variability in how individuals engage with media figures across different platforms and contexts. Additionally, the lower consistency in this dimension may reflect diverse interpretations of digital interaction behaviors across Brazilian subcultures or generational differences in social media usage patterns. The moderate reliability of the Affective dimension suggests some variability in emotional engagement patterns, possibly reflecting cultural heterogeneity in expressing parasocial emotions. The correlation analyses revealed distinct patterns among dimensions, with the Decisional dimension showing the strongest association with overall parasocial engagement, followed by the Cognitive and Affective dimensions. The relatively weaker correlation of the Behavioral dimension with the total score aligns with its lower internal consistency and suggest that, while engagement behaviors are an integral aspect of parasocial relationships, they may be more indirectly shaped by decisional, cognitive, and emotional factors within the Brazilian context.

The replication of the correlated bifactorial structure in Brazil reinforces the theoretical foundations of the MMPR, supporting the view that parasocial relationship engagement is best conceptualized as a multidimensional construct. The presence of a robust general factor suggests that individuals engage with media figures through a shared underlying mechanism of parasocial attachment, while the four distinct dimensions capture unique engagement aspects. These findings align with previous research emphasizing the complexity of parasocial relationships and the need for multidimensional assessment approaches^3,21^, as well as with recent replications of the correlated bifactor model of the MMPR in Hungary, Indonesia, Canada, and Poland^14,15^. The successful structural replication in the Brazilian sample supports the MMPR’s cross-cultural applicability and its potential for comparative studies across different media environments and cultural contexts. Despite overall structural support, notable variations emerged in specific dimension performance. The Affective and Cognitive dimensions demonstrated strong associations with the general factor, reinforcing emotional attachment and cognitive engagement as central components of parasocial relationships^8,22^. The Behavioral dimension showed consistently weaker performance across validity and reliability indices, indicating potential cultural differences in how engagement behaviors, such as liking, commenting, and sharing, are enacted or interpreted in the Brazilian context. While the Decisional dimension exhibited strong psychometric properties, its negative factor loadings suggest complex factorial relationships that may reflect implicit influence processes rather than wording issues. These contrasting dimensional patterns (i.e., behavioral underperformance alongside decisional importance) reveal culturally specific engagement mechanisms that merit detailed discussion.

The Behavioral dimension’s weaker performance suggests that direct engagement behavior (e.g., “liking”, commenting, and sharing media figures’ content) may be less central to parasocial relationships than emotional and cognitive bonds in this sample. While previous research conceptualized parasocial interactions as extending beyond passive media consumption to include active digital engagement ^9,23^, our findings indicate that behavioral parasocial expressions may be more context dependent and shaped by platform-specific norms or cultural (Brazilian in this case) social media usage patterns. Moreover, the MMPR’s Behavioral dimension may inadequately capture contemporary parasocial engagement. For instance, social media algorithms increasingly prioritize passive engagement metrics (e.g., watch time, scrolling behavior, and content retention) over explicit interactions^24–26^. Platforms like YouTube, Instagram, and TikTok use these metrics to personalize content recommendations, with watch time playing a key role in shaping users’ media consumption patterns^26^. Hence, these behaviors may be stronger indicators of sustained parasocial engagement than traditional interaction-based measures. In other words, the underperformance of the MMPR’s Behavioral dimension may reflect broader shifts in user behavior, as parasocial engagement has become increasingly passive. Social media platforms have adapted to these changes by prioritizing metrics of passive over active interactions. Consequently, traditional behavioral indicators included in the MMPR may no longer fully capture how users engage with media figures in contemporary digital environments. Users might regularly watch creators’ content or engage with algorithmically recommended material without ever directly interacting and still develop strong parasocial bonds. The MMPR’s current Behavioral dimension may therefore measure increasingly obsolete engagement patterns. Future research should reconceptualize behavioral engagement to include passive consumption measures, such as viewing frequency, content consistency, or algorithmic responsiveness, that better reflect contemporary user behavior and provide more comprehensive parasocial engagement assessment.

Cross-cultural adaptation studies emphasize the importance of accounting for cultural differences in construct interpretation, as these variations can significantly influence measurement validity and other results^27–29^. The negative factor loadings for the Decisional dimension, despite positive bivariate correlations with the other MMPR dimensions, suggest nuanced relationships between parasocial engagement and perceived decision-making influence. This pattern indicates that while individuals with stronger affective, cognitive, and behavioral engagement also report greater decisional influence in bivariate analyses, this relationship reverses when controlling for general parasocial engagement tendencies. This finding may reflect a distinction between implicit and explicit influence recognition. Research indicates that Brazilians heavily rely on social media for consumer behavior and lifestyle choices^30^, yet the extent to which they consciously acknowledge media figures as shaping their decisions may vary considerably. Cultural norms regarding decision-making autonomy and perceptions of authority could influence self-reported responses, leading to implicit influence that remains unrecognized at the conscious level. Additionally, personality expression in collectivist cultures like Brazil may differ from individualistic contexts, potentially affecting how individuals interpret and report decision-making influence^31,32^. These cultural variations highlight the complexity of measuring parasocial influence across diverse sociocultural contexts and suggest that the Decisional dimension might require cultural adaptation to accurately capture influence in Brazilian populations. Future research should refine the Decisional dimension’s assessment by considering cultural factors in its interpretation and potentially developing culturally adapted items that better capture implicit influence processes. Such refinements would enhance the MMPR’s cross-cultural applicability and provide deeper insights into the mechanisms of parasocial influence in diverse populations.

The overall scale demonstrated strong internal consistency, though dimensional reliability varied considerably. The moderate reliability for the Affective dimension might reflect Brazil’s cultural emphasis on community and interpersonal relationships, where emotional connections to media figures are shaped by social expectations and shared experiences, leading to more diverse response patterns^18^. The Behavioral dimension’s lower reliability likely stems from the dynamic nature of social media engagement in Brazil, where user interactions fluctuate based on platform norms, content trends, and individual preferences^27,33,34^. Brazilians use digital platforms as both social and commercial spaces, creating varied interaction patterns that differ significantly from other cultural contexts^35,36^. Moreover, interactions such as likes and comments may carry different social meanings across Brazilian digital communities, contributing to response inconsistencies and challenging traditional behavioral engagement measures^37–39^. Such cultural variations in psychometric performance have been documented in other cross-cultural adaptations, emphasizing the importance of replication studies to ensure conceptual equivalence across populations^19,40,41^.

The intercorrelation analysis revealed that the Decisional dimension showed the strongest association with overall parasocial engagement, suggesting that perceived influence plays a particularly central role in Brazilian parasocial relationships. This pattern, combined with strong intercorrelations among Affective, Cognitive, and Decisional dimensions, indicates that Brazilian parasocial connections extend beyond emotional attachments to encompass mental engagement and perceived influence on personal decisions^7,42^. The Behavioral dimension’s weaker associations support the perspective that behavioral interactions facilitate rather than define parasocial relationships^23,43^. In the Brazilian context, where social media serves as a dominant space for both personal and commercial interactions, cognitive involvement and perceived influence appear more defining of parasocial bonds than visible engagement behaviors.

The measurement invariance results indicate potential gender differences in how individuals interpret and respond to MMPR items, limiting direct comparisons between groups. The successful model estimation for women, contrasted with computational failures for men, suggests systematic differences in response patterns that warrant investigation. Research indicates that women typically demonstrate greater emotional and cognitive investment in parasocial relationships, experiencing stronger affective connections and engaging in deeper cognitive processing when relating to media figures^11^. The more consistent response patterns observed among women in this study aligns with this higher emotional involvement, whereas men may engage with media figures through different psychological mechanisms or with a less emotionally driven approaches^44,45^. The computational difficulties in the male subsample may reflect inadequate response variance or genuine gender-related differences in factorial structure, rather than sample size issues, as the gender distribution was well-balanced (192 women, 206 men). Future research should systematically explore gendered differences in parasocial engagement, using larger samples, to determine whether men and women may conceptualize parasocial relationships through fundamentally different psychological mechanisms. Particular attention should be paid to whether men tend to engage primarily through behavioral interactions or through alternative psychological processes that are not fully captured by the MMPR or other current measures. Additionally, investigating how these gender patterns vary across context and media platforms would enhance understanding of parasocial dynamics and ensure measurement tools accurately capture diverse engagement styles.

### Implications

This study provides important theoretical insight into the multidimensional nature of parasocial relationships. The successful replication of the bifactor structure reinforces the interconnectedness of Affective, Behavioral, Cognitive, and Decisional dimensions, while revealing cultural-specific patterns. Most notably, the Decisional dimension’s strong psychometric performance highlights the particular salience of perceived influence in Brazilian parasocial relationships, extending beyond the emotional and cognitive engagement emphasized in previous research^7,21^. Moreover, while the Decisional dimension demonstrated strong correlations with the overall MMPR score and the other three subdimensions, its negative loadings in the bifactor model suggest a more complex psychological dynamic. Specifically, these results may indicate that parasocial influence operates through implicit or unconscious mechanisms not fully integrated into individuals’ conscious experience of parasocial engagement. The Decisional items ask respondents to explicitly acknowledge the influence of media figures on their lifestyle, consumption habits, self-presentation, and opinions. However, when modeled alongside general parasocial tendencies, these items behave differently, contributing less to the core construct and even diverging from it. This pattern suggests that individuals may experience real behavioral influence from media figures while remaining partially unaware, reluctant to admit, or psychologically distanced from this influence. In this light, the Decisional dimension’s performance might not reflect measurement error, but instead capture a distinct layer of parasocial engagement, one rooted in implicit decision-making and self-regulatory mechanisms rather than overt emotional or cognitive involvement^46^. If so, this finding warrant further exploration into the unconscious aspects of media influence and suggest that future revisions of the MMPR could benefit from more nuanced items that tap into indirect or automatic processes of influence.

The validated Brazilian Portuguese MMPR provides researchers and practitioners with a psychometrically sound instrument for assessing parasocial relationships engagement in Brazilian populations. This tool offers particular value for social media marketing and consumer behavior research, where understanding the psychological mechanisms of influencer impact can inform engagement strategies and campaign effectiveness. Given the strong performance of the Decisional dimension, practitioners should recognize that media figures may shape consumer preferences and lifestyle choices through subtle influence processes that extend beyond traditional persuasion models. The scale also holds promise for mental health applications, as parasocial relationships may serve as coping mechanisms or emotion-regulation functions, particularly in contexts of loneliness or social isolation^7,22^. However, the current study’s lack of direct coping measures limits conclusions about these therapeutic functions. Future research integrating established coping assessments could more explicitly explore how parasocial engagement serves as an affective and cognitive coping strategy.

### Limitations

Several limitations should be acknowledged. For instance, while the overall model fit was acceptable, the RMSEA indicated a marginally acceptable fit. This, combined with the lower internal consistency of the Affective and Behavioral dimensions, suggests potential limitations in capturing all facets of parasocial engagement in the Brazilian context. These findings align with the complexities often observed in cross-cultural psychometric adaptation and underscore the need for ongoing refinement of the instrument^27^. The Behavioral dimension’s underperformance may reflect the measure’s focus on traditional active engagement behaviors rather than the passive consumption patterns that increasingly characterize contemporary social media use. Future research should consider developing culturally adapted items that capture both active and passive behavioral engagement patterns relevant to Brazilian digital contexts. The virtual snowball sampling approach, while effective for recruitment, precluded collection of regional demographic data. Given Brazil’s substantial regional diversity in social media usage patterns and cultural values, this limitation constrains generalizability. Future studies should employ stratified sampling across Brazilian regions to examine potential geographic variations in parasocial engagement, social media usage, and consumer behavior, thereby ensuring broader representativeness.

The cross-sectional design also limits understanding of parasocial relationship development and temporal dynamics. Longitudinal research is essential to examine how these relationships evolve, their stability over time, and their long-term psychological impacts as protective and risk-related factors, as previously suggested by other researchers^7,47–49^. Additionally, it is important to acknowledge that the original validation study by García and colleagues^3^ used principal component analysis (PCA) with varimax rotation, a method that, while commonly employed in early scale development, may not optimally capture latent constructs due to its focus on data reduction rather than underlying factor modeling. Given this methodological limitation and the recent successful replications of the correlated bifactor model in other cultural contexts^14,15^, we employed CFA to rigorously test the hypothesized correlated bifactor structure within the Brazilian sample. Nevertheless, we acknowledge that confirming the MMPR’s dimensional validity in a new cultural context would benefit from a mixed-method psychometric approach. Specifically, an initial exploratory factor analysis (EFA) could help identify culturally sensitive item functioning, including potential cross-loadings or misfitting items, prior to CFA. We recommend that future research adopt such a sequential approach to further refine the instrument for robust cross-cultural use. Moreover, gender invariance testing proved inconclusive due to model convergence issues in the male subsample, despite balanced gender representation. This limitation prevents definitive conclusions about gender differences in parasocial engagement patterns and highlights the need for targeted investigation of gender-specific measurement models.

Finally, this study focused exclusively on the internal structure of the MMPR and did not examine its external validity through convergent, discriminant, or nomological validation. Prior research has shown that MMPR scores are associated with a range of relevant constructs, including early maladaptive schemas and emotional well-being^15^, social comparison and self-esteem^3,14^, celebrity attachment^14,50^, emotion regulation and the need to belong^51^, identification and affective resonance with fictive TV-characters^48^, as well as personality traits and psychological immersion in social media content^52^. Investigating such associations in Brazilian samples is essential for developing the MMPR’s nomological network, which would clarify its theoretical position and confirm that it measures a distinct yet conceptually meaningful construct. Future research should include related psychological measures to provide a more comprehensive validation and deepen our understanding of the psychological functions and implications of parasocial engagement in culturally diverse contexts like Brazil.

### Conclusion and final remarks

This study makes important contributions to cross-cultural research on parasocial relationships by demonstrating both universal and culturally specific patterns in the MMPR’s performance. The successful replication of the correlated bifactor structure supports the general applicability of the ABCD model of parasocial relationship engagement while revealing distinct cultural features within the Brazilian context. Most notably, the Decisional dimension’s strong psychometric performance alongside negative loadings suggests that perceived influence operates through implicit mechanisms that may be culturally shaped. These findings challenge assumptions of universal parasocial engagement patterns and underscore the necessity of culturally sensitive adaptations that reflect local media consumption behaviors, societal values, and decision-making norms. The variability in dimensional internal consistency, particularly for the Behavioral dimension, highlights how evolving digital practices and cultural contexts influence measurement validity.

The results advance theoretical understandings by illustrating how cultural norms and technological evolution shape parasocial engagement. While emotional and cognitive components remain foundational across cultures, the Brazilian context reveals unique patterns in how individuals experience and report media figure influence. This cultural specificity suggests that future cross-cultural research move beyond simple translation and adaptation toward developing culturally informed theoretical models. The scale’s overall strong reliability confirms its utility for measuring parasocial relationships, yet dimensional variations indicate opportunities for refinement. Future research should focus on developing culturally adapted items, particularly for behavioral engagement measures that capture contemporary social media practices and investigating the implicit influence processes suggested by the Decisional dimension’s complex performance. By incorporating these cultural insights, researchers can develop more nuanced models of parasocial engagement that effectively capture the complexities of digital interactions within diverse and evolving social landscapes.

*“Na televisão*,* nada se cria*,* tudo se copia"*.

*[“On television*,* nothing is created*,* everything is copied.“]*.

*José Abelardo Barbosa de Medeiros (Chacrinha)*.

## Data Availability

The data supporting the findings of this study are available from the research group, but restrictions apply to the availability of these data, and the data are not publicly available. In case data is requested, please contact TMCD and DG.
